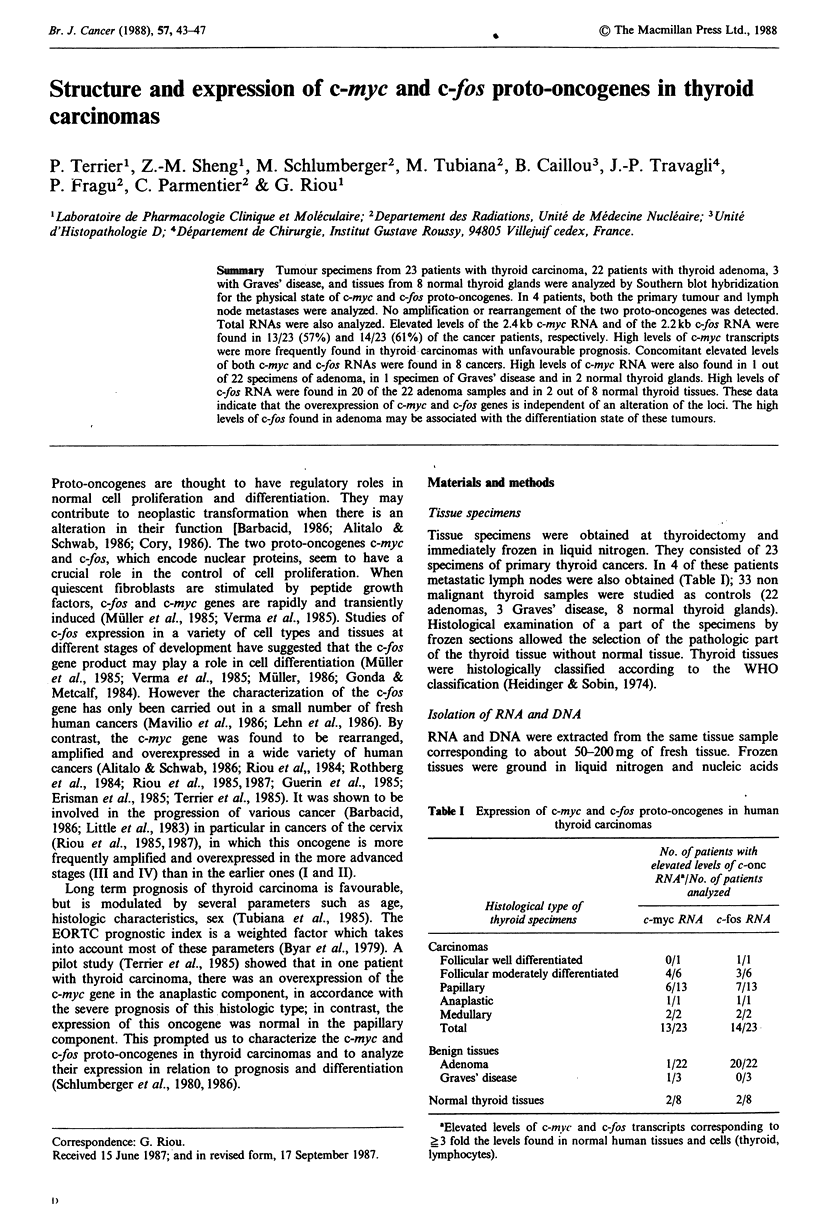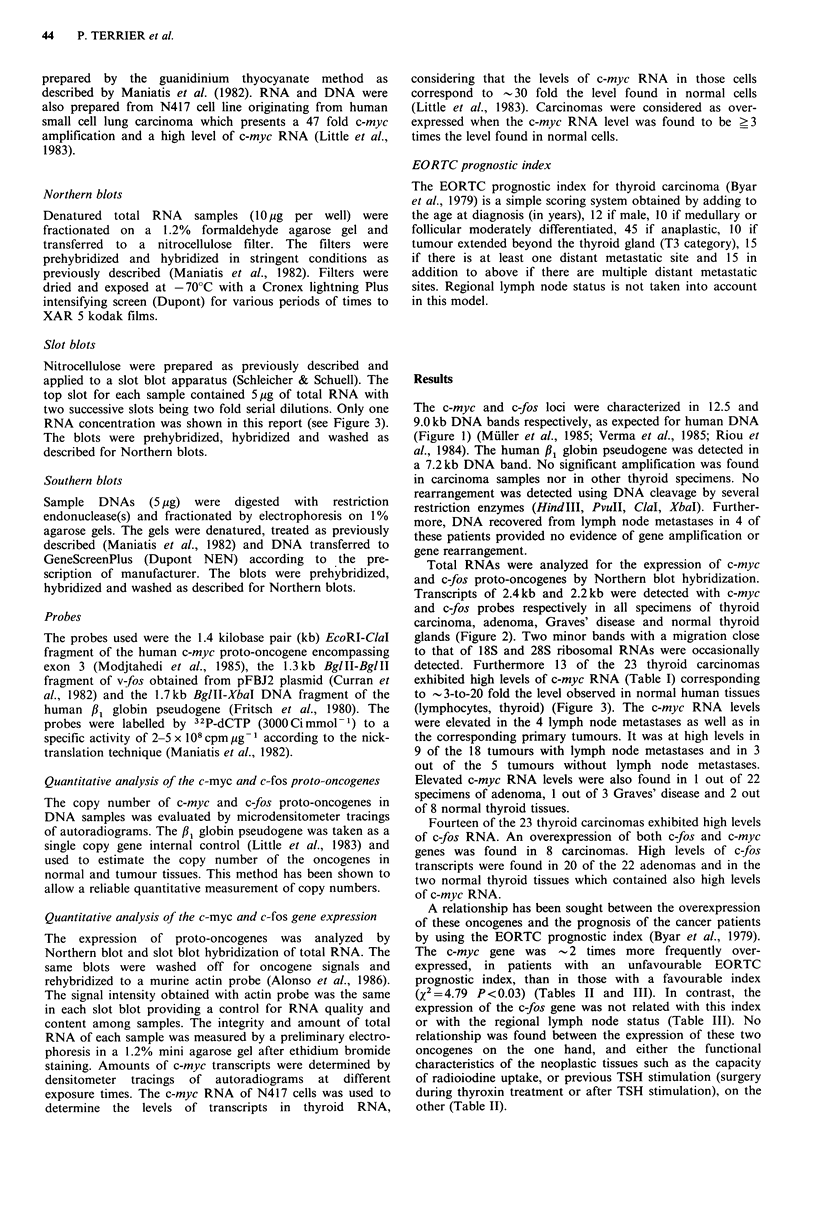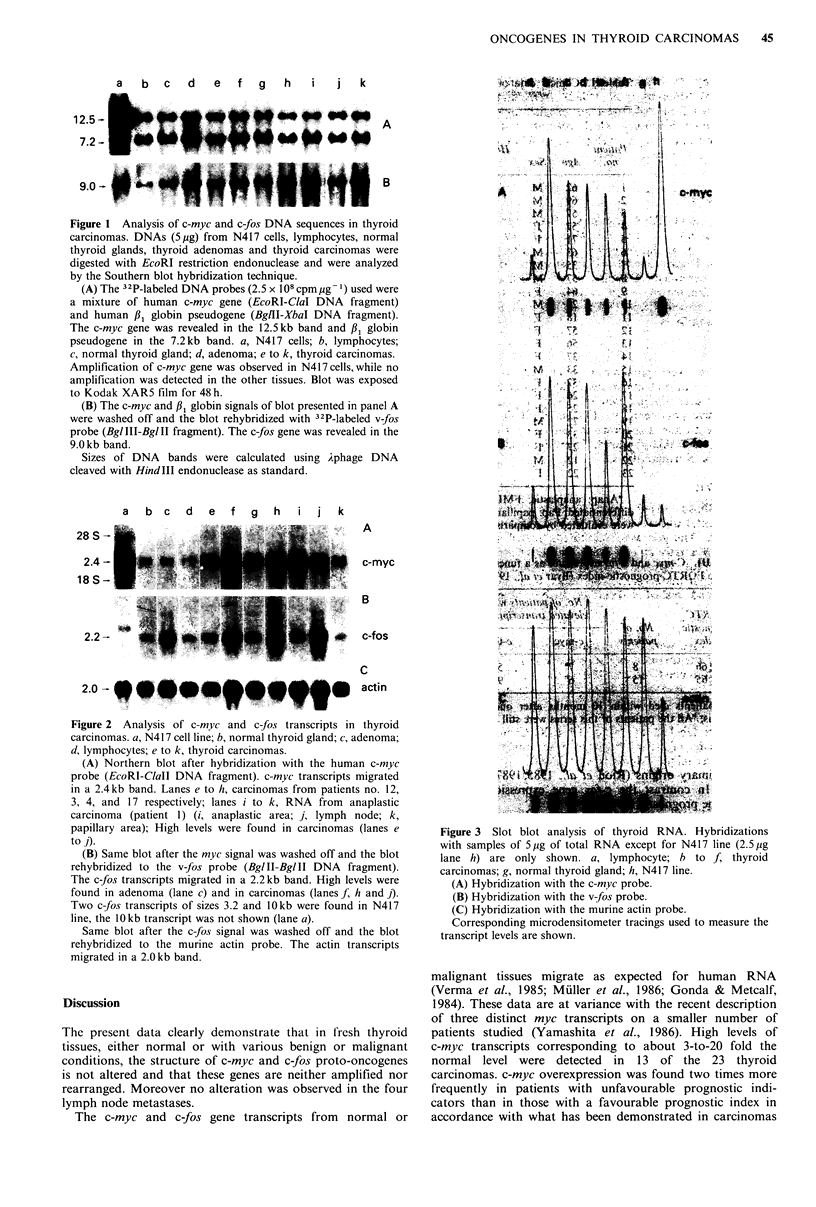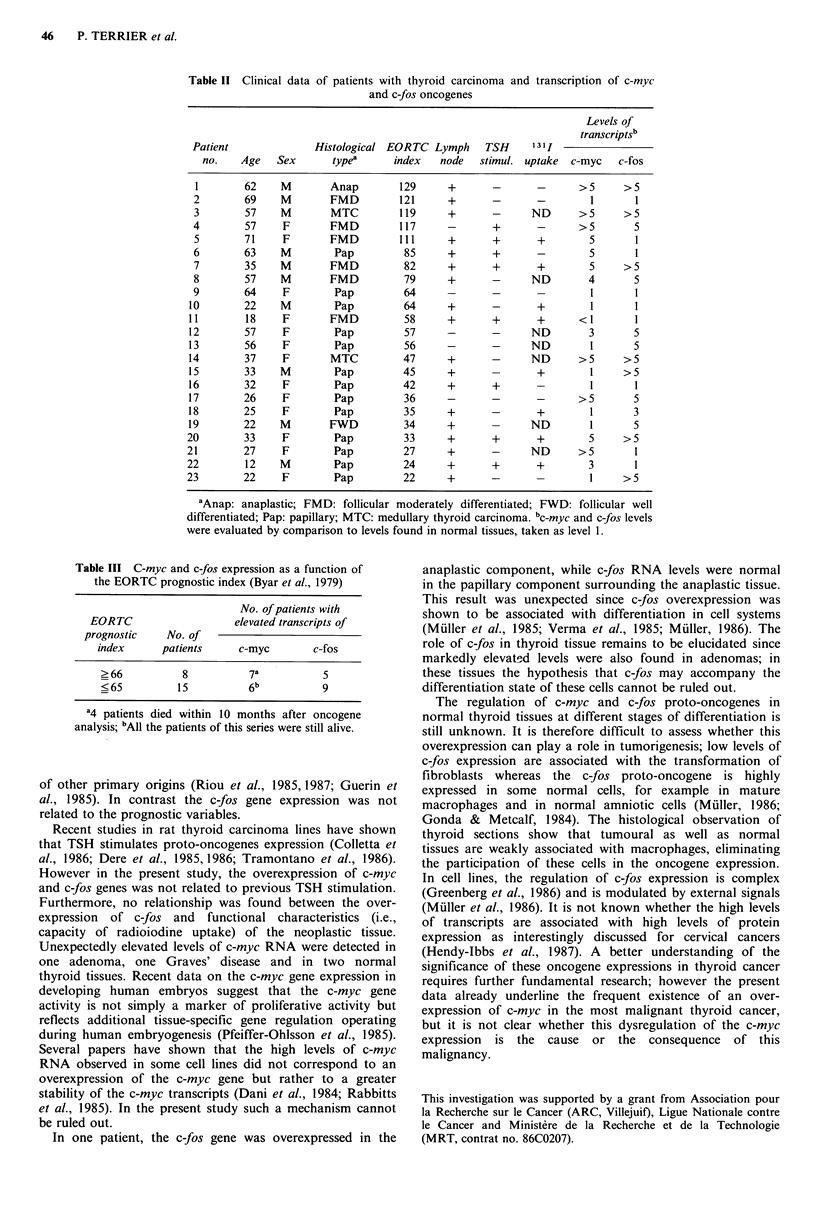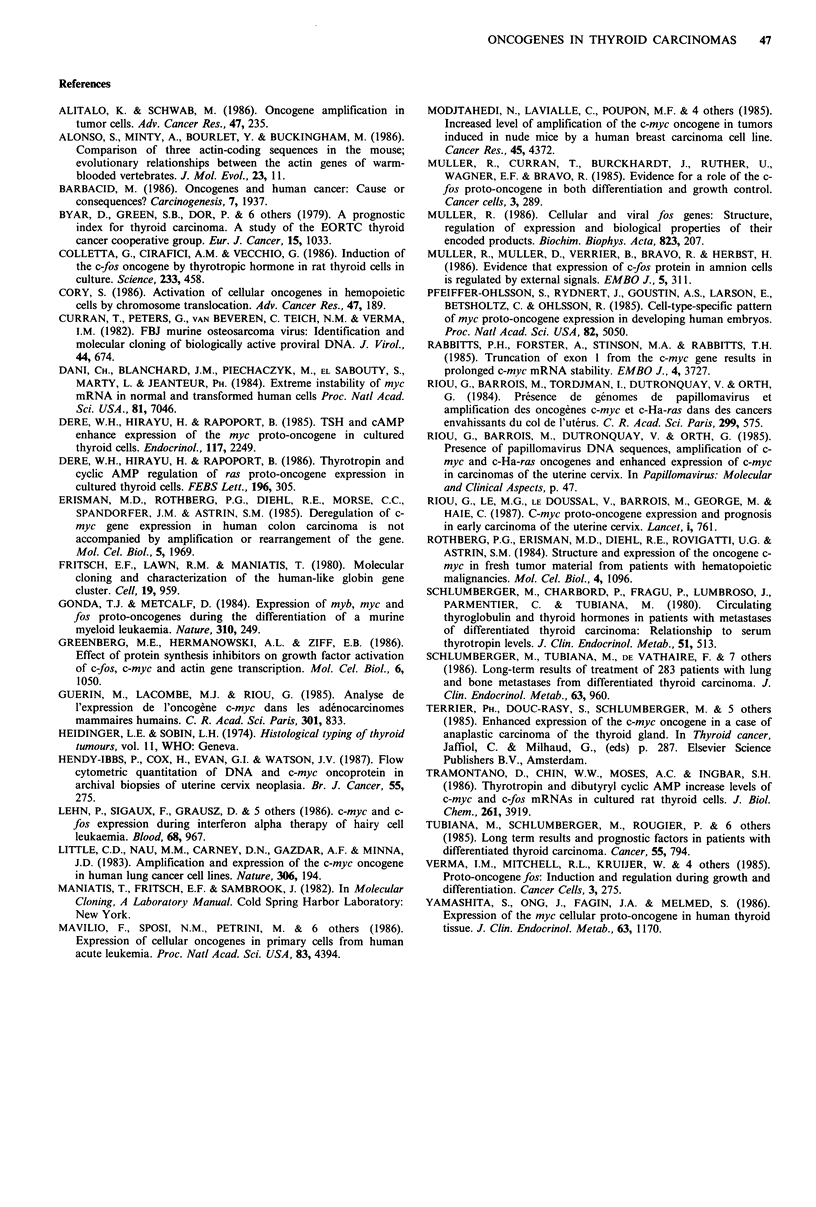# Structure and expression of c-myc and c-fos proto-oncogenes in thyroid carcinomas.

**DOI:** 10.1038/bjc.1988.6

**Published:** 1988-01

**Authors:** P. Terrier, Z. M. Sheng, M. Schlumberger, M. Tubiana, B. Caillou, J. P. Travagli, P. Fragu, C. Parmentier, G. Riou

**Affiliations:** Laboratoire de Pharmacologie Clinique et Moléculaire, Institut Gustave Roussy, Villejuif, France.

## Abstract

**Images:**


*Br. J. Cancer* (1988), **57**, 43–47 © The Macmillan Press Ltd., 1988

### Structure and expression of c-*myc* and c-*fos* proto-oncogenes in thyroid carcinomas

P. Terrier[1], Z.-M. Sheng[1], M. Schlumberger[2], M. Tubiana[2], B. Caillou[3], J.-P. Travagli[4], P. Fragu[2], C. Parmentier[2] & G. Riou[1]

[1]*Laboratoire de Pharmacologie Clinique et Moléculaire;* [2]*Departement des Radiations, Unité de Médecine Nucléaire;* [3]*Unité d'Histopathologie D;* [4]*Département de Chirurgie, Institut Gustave Roussy, 94805 Villejuif cedex, France.*

**Summary** Tumour specimens from 23 patients with thyroid carcinoma, 22 patients with thyroid adenoma, 3 with Graves' disease, and tissues from 8 normal thyroid glands were analyzed by Southern blot hybridization for the physical state of c-*myc* and c-*fos* proto-oncogenes. In 4 patients, both the primary tumour and lymph node metastases were analyzed. No amplification or rearrangement of the two proto-oncogenes was detected. Total RNAs were also analyzed. Elevated levels of the 2.4 kb c-*myc* RNA and of the 2.2 kb c-*fos* RNA were found in 13/23 (57%) and 14/23 (61%) of the cancer patients, respectively. High levels of c-*myc* transcripts were more frequently found in thyroid carcinomas with unfavourable prognosis. Concomitant elevated levels of both c-*myc* and c-*fos* RNAs were found in 8 cancers. High levels of c-*myc* RNA were also found in 1 out of 22 specimens of adenoma, in 1 specimen of Graves' disease and in 2 normal thyroid glands. High levels of c-*fos* RNA were found in 20 of the 22 adenoma samples and in 2 out of 8 normal thyroid tissues. These data indicate that the overexpression of c-*myc* and c-*fos* genes is independent of an alteration of the loci. The high levels of c-*fos* found in adenoma may be associated with the differentiation state of these tumours.

Proto-oncogenes are thought to have regulatory roles in normal cell proliferation and differentiation. They may contribute to neoplastic transformation when there is an alteration in their function [Barbacid, 1986; Alitalo & Schwab, 1986; Cory, 1986). The two proto-oncogenes c-*myc* and c-*fos*, which encode nuclear proteins, seem to have a crucial role in the control of cell proliferation. When quiescent fibroblasts are stimulated by peptide growth factors, c-*fos* and c-*myc* genes are rapidly and transiently induced (Müller *et al.*, 1985; Verma *et al.*, 1985). Studies of c-*fos* expression in a variety of cell types and tissues at different stages of development have suggested that the c-*fos* gene product may play a role in cell differentiation (Müller *et al.*, 1985; Verma *et al.*, 1985; Müller, 1986; Gonda & Metcalf, 1984). However the characterization of the c-*fos* gene has only been carried out in a small number of fresh human cancers (Mavilio *et al.*, 1986; Lehn *et al.*, 1986). By contrast, the c-*myc* gene was found to be rearranged, amplified and overexpressed in a wide variety of human cancers (Alitalo & Schwab, 1986; Riou *et al*,, 1984; Rothberg *et al.*, 1984; Riou *et al.*, 1985, 1987; Guerin *et al.*, 1985; Erisman *et al.*, 1985; Terrier *et al.*, 1985). It was shown to be involved in the progression of various cancer (Barbacid, 1986; Little *et al.*, 1983) in particular in cancers of the cervix (Riou *et al.*, 1985, 1987), in which this oncogene is more frequently amplified and overexpressed in the more advanced stages (III and IV) than in the earlier ones (I and II).

Long term prognosis of thyroid carcinoma is favourable, but is modulated by several parameters such as age, histologic characteristics, sex (Tubiana *et al.*, 1985). The EORTC prognostic index is a weighted factor which takes into account most of these parameters (Byar *et al.*, 1979). A pilot study (Terrier *et al.*, 1985) showed that in one patient with thyroid carcinoma, there was an overexpression of the c-*myc* gene in the anaplastic component, in accordance with the severe prognosis of this histologic type; in contrast, the expression of this oncogene was normal in the papillary component. This prompted us to characterize the c-*myc* and c-*fos* proto-oncogenes in thyroid carcinomas and to analyze their expression in relation to prognosis and differentiation (Schlumberger *et al.*, 1980, 1986).

Correspondence: G. Riou.
Received 15 June 1987; and in revised form, 17 September 1987.

### Materials and methods

### *Tissue specimens*

Tissue specimens were obtained at thyroidectomy and immediately frozen in liquid nitrogen. They consisted of 23 specimens of primary thyroid cancers. In 4 of these patients metastatic lymph nodes were also obtained (Table I); 33 non malignant thyroid samples were studied as controls (22 adenomas, 3 Graves' disease, 8 normal thyroid glands). Histological examination of a part of the specimens by frozen sections allowed the selection of the pathologic part of the thyroid tissue without normal tissue. Thyroid tissues were histologically classified according to the WHO classification (Heidinger & Sobin, 1974).

### *Isolation of RNA and DNA*

RNA and DNA were extracted from the same tissue sample corresponding to about 50–200 mg of fresh tissue. Frozen tissues were ground in liquid nitrogen and nucleic acids

**Table I** Expression of c-*myc* and c-*fos* proto-oncogenes in human thyroid carcinomas

| *Histological type of thyroid specimens* | *No. of patients with elevated levels of c-onc RNA[a]/No. of patients analyzed* | |
|---|---|---|
| | c-myc *RNA* | c-fos *RNA* |
| Carcinomas | | |
| Follicular well differentiated | 0/1 | 1/1 |
| Follicular moderately differentiated | 4/6 | 3/6 |
| Papillary | 6/13 | 7/13 |
| Anaplastic | 1/1 | 1/1 |
| Medullary | 2/2 | 2/2 |
| Total | 13/23 | 14/23 |
| Benign tissues | | |
| Adenoma | 1/22 | 20/22 |
| Graves' disease | 1/3 | 0/3 |
| Normal thyroid tissues | 2/8 | 2/8 |

[a]Elevated levels of c-*myc* and c-*fos* transcripts corresponding to ≥3 fold the levels found in normal human tissues and cells (thyroid, lymphocytes).

prepared by the guanidinium thyocyanate method as described by Maniatis *et al.* (1982). RNA and DNA were also prepared from N417 cell line originating from human small cell lung carcinoma which presents a 47 fold c-*myc* amplification and a high level of c-*myc* RNA (Little *et al.*, 1983).

*Northern blots*

Denatured total RNA samples (10 $\mu$g per well) were fractionated on a 1.2% formaldehyde agarose gel and transferred to a nitrocellulose filter. The filters were prehybridized and hybridized in stringent conditions as previously described (Maniatis *et al.*, 1982). Filters were dried and exposed at $-70°C$ with a Cronex lightning Plus intensifying screen (Dupont) for various periods of times to XAR 5 kodak films.

*Slot blots*

Nitrocellulose were prepared as previously described and applied to a slot blot apparatus (Schleicher & Schuell). The top slot for each sample contained 5 $\mu$g of total RNA with two successive slots being two fold serial dilutions. Only one RNA concentration was shown in this report (see Figure 3). The blots were prehybridized, hybridized and washed as described for Northern blots.

*Southern blots*

Sample DNAs (5 $\mu$g) were digested with restriction endonuclease(s) and fractionated by electrophoresis on 1% agarose gels. The gels were denatured, treated as previously described (Maniatis *et al.*, 1982) and DNA transferred to GeneScreenPlus (Dupont NEN) according to the prescription of manufacturer. The blots were prehybridized, hybridized and washed as described for Northern blots.

*Probes*

The probes used were the 1.4 kilobase pair (kb) *Eco*RI-*Cla*I fragment of the human c-*myc* proto-oncogene encompassing exon 3 (Modjtahedi *et al.*, 1985), the 1.3 kb *Bgl*II-*Bgl*II fragment of v-*fos* obtained from pFBJ2 plasmid (Curran *et al.*, 1982) and the 1.7 kb *Bgl*II-*Xba*I DNA fragment of the human $\beta_1$ globin pseudogene (Fritsch *et al.*, 1980). The probes were labelled by $^{32}$P-dCTP (3000 Ci mmol$^{-1}$) to a specific activity of $2–5 \times 10^8$ cpm $\mu g^{-1}$ according to the nick-translation technique (Maniatis *et al.*, 1982).

*Quantitative analysis of the c-myc and c-fos proto-oncogenes*

The copy number of c-*myc* and c-*fos* proto-oncogenes in DNA samples was evaluated by microdensitometer tracings of autoradiograms. The $\beta_1$ globin pseudogene was taken as a single copy gene internal control (Little *et al.*, 1983) and used to estimate the copy number of the oncogenes in normal and tumour tissues. This method has been shown to allow a reliable quantitative measurement of copy numbers.

*Quantitative analysis of the c-myc and c-fos gene expression*

The expression of proto-oncogenes was analyzed by Northern blot and slot blot hybridization of total RNA. The same blots were washed off for oncogene signals and rehybridized to a murine actin probe (Alonso *et al.*, 1986). The signal intensity obtained with actin probe was the same in each slot blot providing a control for RNA quality and content among samples. The integrity and amount of total RNA of each sample was measured by a preliminary electrophoresis in a 1.2% mini agarose gel after ethidium bromide staining. Amounts of c-*myc* transcripts were determined by densitometer tracings of autoradiograms at different exposure times. The c-*myc* RNA of N417 cells was used to determine the levels of transcripts in thyroid RNA, considering that the levels of c-*myc* RNA in those cells correspond to $\sim$30 fold the level found in normal cells (Little *et al.*, 1983). Carcinomas were considered as overexpressed when the c-*myc* RNA level was found to be $\geq$3 times the level found in normal cells.

*EORTC prognostic index*

The EORTC prognostic index for thyroid carcinoma (Byar *et al.*, 1979) is a simple scoring system obtained by adding to the age at diagnosis (in years), 12 if male, 10 if medullary or follicular moderately differentiated, 45 if anaplastic, 10 if tumour extended beyond the thyroid gland (T3 category), 15 if there is at least one distant metastatic site and 15 in addition to above if there are multiple distant metastatic sites. Regional lymph node status is not taken into account in this model.

### Results

The c-*myc* and c-*fos* loci were characterized in 12.5 and 9.0 kb DNA bands respectively, as expected for human DNA (Figure 1) (Müller *et al.*, 1985; Verma *et al.*, 1985; Riou *et al.*, 1984). The human $\beta_1$ globin pseudogene was detected in a 7.2 kb DNA band. No significant amplification was found in carcinoma samples nor in other thyroid specimens. No rearrangement was detected using DNA cleavage by several restriction enzymes (*Hind*III, *Pvu*II, *Cla*I, *Xba*I). Furthermore, DNA recovered from lymph node metastases in 4 of these patients provided no evidence of gene amplification or gene rearrangement.

Total RNAs were analyzed for the expression of c-*myc* and c-*fos* proto-oncogenes by Northern blot hybridization. Transcripts of 2.4 kb and 2.2 kb were detected with c-*myc* and c-*fos* probes respectively in all specimens of thyroid carcinoma, adenoma, Graves' disease and normal thyroid glands (Figure 2). Two minor bands with a migration close to that of 18S and 28S ribosomal RNAs were occasionally detected. Furthermore 13 of the 23 thyroid carcinomas exhibited high levels of c-*myc* RNA (Table I) corresponding to $\sim$3-to-20 fold the level observed in normal human tissues (lymphocytes, thyroid) (Figure 3). The c-*myc* RNA levels were elevated in the 4 lymph node metastases as well as in the corresponding primary tumours. It was at high levels in 9 of the 18 tumours with lymph node metastases and in 3 out of the 5 tumours without lymph node metastases. Elevated c-*myc* RNA levels were also found in 1 out of 22 specimens of adenoma, 1 out of 3 Graves' disease and 2 out of 8 normal thyroid tissues.

Fourteen of the 23 thyroid carcinomas exhibited high levels of c-*fos* RNA. An overexpression of both c-*fos* and c-*myc* genes was found in 8 carcinomas. High levels of c-*fos* transcripts were found in 20 of the 22 adenomas and in the two normal thyroid tissues which contained also high levels of c-*myc* RNA.

A relationship has been sought between the overexpression of these oncogenes and the prognosis of the cancer patients by using the EORTC prognostic index (Byar *et al.*, 1979). The c-*myc* gene was $\sim$2 times more frequently overexpressed, in patients with an unfavourable EORTC prognostic index, than in those with a favourable index ($\chi^2 = 4.79$ $P < 0.03$) (Tables II and III). In contrast, the expression of the c-*fos* gene was not related with this index or with the regional lymph node status (Table III). No relationship was found between the expression of these two oncogenes on the one hand, and either the functional characteristics of the neoplastic tissues such as the capacity of radioiodine uptake, or previous TSH stimulation (surgery during thyroxin treatment or after TSH stimulation), on the other (Table II).

**Figure 1** Analysis of c-*myc* and c-*fos* DNA sequences in thyroid carcinomas. DNAs (5 $\mu$g) from N417 cells, lymphocytes, normal thyroid glands, thyroid adenomas and thyroid carcinomas were digested with *Eco*RI restriction endonuclease and were analyzed by the Southern blot hybridization technique.

(**A**) The $^{32}$P-labeled DNA probes (2.5 x $10^8$ cpm $\mu g^{-1}$) used were a mixture of human c-*myc* gene (*Eco*RI-*Cla*I DNA fragment) and human $\beta_1$ globin pseudogene (*Bgl*II-*Xba*I DNA fragment). The c-*myc* gene was revealed in the 12.5 kb band and $\beta_1$ globin pseudogene in the 7.2 kb band. *a*, N417 cells; *b*, lymphocytes; *c*, normal thyroid gland; *d*, adenoma; *e* to *k*, thyroid carcinomas. Amplification of c-*myc* gene was observed in N417 cells, while no amplification was detected in the other tissues. Blot was exposed to Kodak XAR5 film for 48 h.

(**B**) The c-*myc* and $\beta_1$ globin signals of blot presented in panel **A** were washed off and the blot rehybridized with $^{32}$P-labeled v-*fos* probe (*Bgl*III-*Bgl*II fragment). The c-*fos* gene was revealed in the 9.0 kb band.

Sizes of DNA bands were calculated using $\lambda$phage DNA cleaved with *Hind*III endonuclease as standard.

**Figure 2** Analysis of c-*myc* and c-*fos* transcripts in thyroid carcinomas. *a*, N417 cell line; *b*, normal thyroid gland; *c*, adenoma; *d*, lymphocytes; *e* to *k*, thyroid carcinomas.

(**A**) Northern blot after hybridization with the human c-*myc* probe (*Eco*RI-*Cla*II DNA fragment). c-*myc* transcripts migrated in a 2.4 kb band. Lanes *e* to *h*, carcinomas from patients no. 12, 3, 4, and 17 respectively; lanes *i* to *k*, RNA from anaplastic carcinoma (patient 1) (*i*, anaplastic area; *j*, lymph node; *k*, papillary area); High levels were found in carcinomas (lanes *e* to *j*).

(**B**) Same blot after the *myc* signal was washed off and the blot rehybridized to the v-*fos* probe (*Bgl*II-*Bgl*II DNA fragment). The c-*fos* transcripts migrated in a 2.2 kb band. High levels were found in adenoma (lane *c*) and in carcinomas (lanes *f*, *h* and *j*). Two c-*fos* transcripts of sizes 3.2 and 10 kb were found in N417 line, the 10 kb transcript was not shown (lane *a*).

Same blot after the c-*fos* signal was washed off and the blot rehybridized to the murine actin probe. The actin transcripts migrated in a 2.0 kb band.

**Figure 3** Slot blot analysis of thyroid RNA. Hybridizations with samples of 5 $\mu$g of total RNA except for N417 line (2.5 $\mu$g lane *h*) are only shown. *a*, lymphocyte; *b* to *f*, thyroid carcinomas; *g*, normal thyroid gland; *h*, N417 line.

(**A**) Hybridization with the c-*myc* probe.

(**B**) Hybridization with the v-*fos* probe.

(**C**) Hybridization with the murine actin probe.

Corresponding microdensitometer tracings used to measure the transcript levels are shown.

### Discussion

The present data clearly demonstrate that in fresh thyroid tissues, either normal or with various benign or malignant conditions, the structure of c-*myc* and c-*fos* proto-oncogenes is not altered and that these genes are neither amplified nor rearranged. Moreover no alteration was observed in the four lymph node metastases.

The c-*myc* and c-*fos* gene transcripts from normal or malignant tissues migrate as expected for human RNA (Verma *et al.*, 1985; Müller *et al.*, 1986; Gonda & Metcalf, 1984). These data are at variance with the recent description of three distinct *myc* transcripts on a smaller number of patients studied (Yamashita *et al.*, 1986). High levels of c-*myc* transcripts corresponding to about 3-to-20 fold the normal level were detected in 13 of the 23 thyroid carcinomas. c-*myc* overexpression was found two times more frequently in patients with unfavourable prognostic indicators than in those with a favourable prognostic index in accordance with what has been demonstrated in carcinomas

**Table II** Clinical data of patients with thyroid carcinoma and transcription of c-*myc* and c-*fos* oncogenes

| *Patient no.* | *Age* | *Sex* | *Histological type*[a] | *EORTC index* | *Lymph node* | *TSH stimul.* | $^{131}I$ *uptake* | *Levels of transcripts*[b] c-myc | c-fos |
|---|---|---|---|---|---|---|---|---|---|
| 1 | 62 | M | Anap | 129 | + | − | − | >5 | >5 |
| 2 | 69 | M | FMD | 121 | + | − | − | 1 | 1 |
| 3 | 57 | M | MTC | 119 | + | − | ND | >5 | >5 |
| 4 | 57 | F | FMD | 117 | − | + | − | >5 | 5 |
| 5 | 71 | F | FMD | 111 | + | + | + | 5 | 1 |
| 6 | 63 | M | Pap | 85 | + | + | − | 5 | 1 |
| 7 | 35 | M | FMD | 82 | + | + | + | 5 | >5 |
| 8 | 57 | M | FMD | 79 | + | − | ND | 4 | 5 |
| 9 | 64 | F | Pap | 64 | − | − | − | 1 | 1 |
| 10 | 22 | M | Pap | 64 | + | − | + | 1 | 1 |
| 11 | 18 | F | FMD | 58 | + | + | + | <1 | 1 |
| 12 | 57 | F | Pap | 57 | − | − | ND | 3 | 5 |
| 13 | 56 | F | Pap | 56 | − | − | ND | 1 | 5 |
| 14 | 37 | F | MTC | 47 | + | − | ND | >5 | >5 |
| 15 | 33 | M | Pap | 45 | + | − | + | 1 | >5 |
| 16 | 32 | F | Pap | 42 | + | + | − | 1 | 1 |
| 17 | 26 | F | Pap | 36 | − | − | − | >5 | 5 |
| 18 | 25 | F | Pap | 35 | + | − | + | 1 | 3 |
| 19 | 22 | M | FWD | 34 | + | − | ND | 1 | 5 |
| 20 | 33 | F | Pap | 33 | + | + | + | 5 | >5 |
| 21 | 27 | F | Pap | 27 | + | − | ND | >5 | 1 |
| 22 | 12 | M | Pap | 24 | + | + | + | 3 | 1 |
| 23 | 22 | F | Pap | 22 | + | − | − | 1 | >5 |

[a]Anap: anaplastic; FMD: follicular moderately differentiated; FWD: follicular well differentiated; Pap: papillary; MTC: medullary thyroid carcinoma. [b]c-*myc* and c-*fos* levels were evaluated by comparison to levels found in normal tissues, taken as level 1.

**Table III** C-*myc* and c-*fos* expression as a function of the EORTC prognostic index (Byar *et al.*, 1979)

| *EORTC prognostic index* | *No. of patients* | *No. of patients with elevated transcripts of* c-myc | c-fos |
|---|---|---|---|
| ≧66 | 8 | 7[a] | 5 |
| ≦65 | 15 | 6[b] | 9 |

[a]4 patients died within 10 months after oncogene analysis; [b]All the patients of this series were still alive.

of other primary origins (Riou *et al.*, 1985, 1987; Guerin *et al.*, 1985). In contrast the c-*fos* gene expression was not related to the prognostic variables.

Recent studies in rat thyroid carcinoma lines have shown that TSH stimulates proto-oncogenes expression (Colletta *et al.*, 1986; Dere *et al.*, 1985, 1986; Tramontano *et al.*, 1986). However in the present study, the overexpression of c-*myc* and c-*fos* genes was not related to previous TSH stimulation. Furthermore, no relationship was found between the overexpression of c-*fos* and functional characteristics (i.e., capacity of radioiodine uptake) of the neoplastic tissue. Unexpectedly elevated levels of c-*myc* RNA were detected in one adenoma, one Graves' disease and in two normal thyroid tissues. Recent data on the c-*myc* gene expression in developing human embryos suggest that the c-*myc* gene activity is not simply a marker of proliferative activity but reflects additional tissue-specific gene regulation operating during human embryogenesis (Pfeiffer-Ohlsson *et al.*, 1985). Several papers have shown that the high levels of c-*myc* RNA observed in some cell lines did not correspond to an overexpression of the c-*myc* gene but rather to a greater stability of the c-*myc* transcripts (Dani *et al.*, 1984; Rabbitts *et al.*, 1985). In the present study such a mechanism cannot be ruled out.

In one patient, the c-*fos* gene was overexpressed in the anaplastic component, while c-*fos* RNA levels were normal in the papillary component surrounding the anaplastic tissue. This result was unexpected since c-*fos* overexpression was shown to be associated with differentiation in cell systems (Müller *et al.*, 1985; Verma *et al.*, 1985; Müller, 1986). The role of c-*fos* in thyroid tissue remains to be elucidated since markedly elevated levels were also found in adenomas; in these tissues the hypothesis that c-*fos* may accompany the differentiation state of these cells cannot be ruled out.

The regulation of c-*myc* and c-*fos* proto-oncogenes in normal thyroid tissues at different stages of differentiation is still unknown. It is therefore difficult to assess whether this overexpression can play a role in tumorigenesis; low levels of c-*fos* expression are associated with the transformation of fibroblasts whereas the c-*fos* proto-oncogene is highly expressed in some normal cells, for example in mature macrophages and in normal amniotic cells (Müller, 1986; Gonda & Metcalf, 1984). The histological observation of thyroid sections show that tumoural as well as normal tissues are weakly associated with macrophages, eliminating the participation of these cells in the oncogene expression. In cell lines, the regulation of c-*fos* expression is complex (Greenberg *et al.*, 1986) and is modulated by external signals (Müller *et al.*, 1986). It is not known whether the high levels of transcripts are associated with high levels of protein expression as interestingly discussed for cervical cancers (Hendy-Ibbs *et al.*, 1987). A better understanding of the significance of these oncogene expressions in thyroid cancer requires further fundamental research; however the present data already underline the frequent existence of an overexpression of c-*myc* in the most malignant thyroid cancer, but it is not clear whether this dysregulation of the c-*myc* expression is the cause or the consequence of this malignancy.

This investigation was supported by a grant from Association pour la Recherche sur le Cancer (ARC, Villejuif), Ligue Nationale contre le Cancer and Ministère de la Recherche et de la Technologie (MRT, contrat no. 86C0207).

**References**

ALITALO, K. & SCHWAB, M. (1986). Oncogene amplification in tumor cells. *Adv. Cancer Res.*, **47**, 235.

ALONSO, S., MINTY, A., BOURLET, Y. & BUCKINGHAM, M. (1986). Comparison of three actin-coding sequences in the mouse; evolutionary relationships between the actin genes of warm-blooded vertebrates. *J. Mol. Evol.*, **23**, 11.

BARBACID, M. (1986). Oncogenes and human cancer: Cause or consequences? *Carcinogenesis*, **7**, 1937.

BYAR, D., GREEN, S.B., DOR, P. & 6 others (1979). A prognostic index for thyroid carcinoma. A study of the EORTC thyroid cancer cooperative group. *Eur. J. Cancer*, **15**, 1033.

COLLETTA, G., CIRAFICI, A.M. & VECCHIO, G. (1986). Induction of the c-*fos* oncogene by thyrotropic hormone in rat thyroid cells in culture. *Science*, **233**, 458.

CORY, S. (1986). Activation of cellular oncogenes in hemopoietic cells by chromosome translocation. *Adv. Cancer Res.*, **47**, 189.

CURRAN, T., PETERS, G., VAN BEVEREN, C. TEICH, N.M. & VERMA, I.M. (1982). FBJ murine osteosarcoma virus: Identification and molecular cloning of biologically active proviral DNA. *J. Virol.*, **44**, 674.

DANI, CH., BLANCHARD, J.M., PIECHACZYK, M., EL SABOUTY, S., MARTY, L. & JEANTEUR, PH. (1984). Extreme instability of *myc* mRNA in normal and transformed human cells *Proc. Natl Acad. Sci. USA.*, **81**, 7046.

DERE, W.H., HIRAYU, H. & RAPOPORT, B. (1985). TSH and cAMP enhance expression of the *myc* proto-oncogene in cultured thyroid cells. *Endocrinol.*, **117**, 2249.

DERE, W.H., HIRAYU, H. & RAPOPORT, B. (1986). Thyrotropin and cyclic AMP regulation of *ras* proto-oncogene expression in cultured thyroid cells. *FEBS Lett.*, **196**, 305.

ERISMAN, M.D., ROTHBERG, P.G., DIEHL, R.E., MORSE, C.C., SPANDORFER, J.M. & ASTRIN, S.M. (1985). Deregulation of c-*myc* gene expression in human colon carcinoma is not accompanied by amplification or rearrangement of the gene. *Mol. Cel. Biol.*, **5**, 1969.

FRITSCH, E.F., LAWN, R.M. & MANIATIS, T. (1980). Molecular cloning and characterization of the human-like globin gene cluster. *Cell*, **19**, 959.

GONDA, T.J. & METCALF, D. (1984). Expression of *myb*, *myc* and *fos* proto-oncogenes during the differentiation of a murine myeloid leukaemia. *Nature*, **310**, 249.

GREENBERG, M.E., HERMANOWSKI, A.L. & ZIFF, E.B. (1986). Effect of protein synthesis inhibitors on growth factor activation of c-*fos*, c-*myc* and actin gene transcription. *Mol. Cel. Biol.*, **6**, 1050.

GUERIN, M., LACOMBE, M.J. & RIOU, G. (1985). Analyse de l'expression de l'oncogène c-*myc* dans les adénocarcinomes mammaires humains. *C. R. Acad. Sci. Paris*, **301**, 833.

HEIDINGER, L.E. & SOBIN, L.H. (1974). *Histological typing of thyroid tumours*, vol. 11, WHO: Geneva.

HENDY-IBBS, P., COX, H., EVAN, G.I. & WATSON, J.V. (1987). Flow cytometric quantitation of DNA and c-*myc* oncoprotein in archival biopsies of uterine cervix neoplasia. *Br. J. Cancer*, **55**, 275.

LEHN, P., SIGAUX, F., GRAUSZ, D. & 5 others (1986). c-*myc* and c-*fos* expression during interferon alpha therapy of hairy cell leukaemia. *Blood*, **68**, 967.

LITTLE, C.D., NAU, M.M., CARNEY, D.N., GAZDAR, A.F. & MINNA, J.D. (1983). Amplification and expression of the c-*myc* oncogene in human lung cancer cell lines. *Nature*, **306**, 194.

MANIATIS, T., FRITSCH, E.F. & SAMBROOK, J. (1982). In *Molecular Cloning, A Laboratory Manual.* Cold Spring Harbor Laboratory: New York.

MAVILIO, F., SPOSI, N.M., PETRINI, M. & 6 others (1986). Expression of cellular oncogenes in primary cells from human acute leukemia. *Proc. Natl Acad. Sci. USA*, **83**, 4394.

MODJTAHEDI, N., LAVIALLE, C., POUPON, M.F. & 4 others (1985). Increased level of amplification of the c-*myc* oncogene in tumors induced in nude mice by a human breast carcinoma cell line. *Cancer Res.*, **45**, 4372.

MULLER, R., CURRAN, T., BURCKHARDT, J., RUTHER, U., WAGNER, E.F. & BRAVO, R. (1985). Evidence for a role of the c-*fos* proto-oncogene in both differentiation and growth control. *Cancer cells*, **3**, 289.

MULLER, R. (1986). Cellular and viral *fos* genes: Structure, regulation of expression and biological properties of their encoded products. *Biochim. Biophys. Acta*, **823**, 207.

MULLER, R., MULLER, D., VERRIER, B., BRAVO, R. & HERBST, H. (1986). Evidence that expression of c-*fos* protein in amnion cells is regulated by external signals. *EMBO J.*, **5**, 311.

PFEIFFER-OHLSSON, S., RYDNERT, J., GOUSTIN, A.S., LARSON, E., BETSHOLTZ, C. & OHLSSON, R. (1985). Cell-type-specific pattern of *myc* proto-oncogene expression in developing human embryos. *Proc. Natl Acad. Sci. USA*, **82**, 5050.

RABBITTS, P.H., FORSTER, A., STINSON, M.A. & RABBITTS, T.H. (1985). Truncation of exon 1 from the c-*myc* gene results in prolonged c-*myc* mRNA stability. *EMBO J.*, **4**, 3727.

RIOU, G., BARROIS, M., TORDJMAN, I., DUTRONQUAY, V. & ORTH, G. (1984). Présence de génomes de papillomavirus et amplification des oncogènes c-*myc* et c-Ha-*ras* dans des cancers envahissants du col de l'utérus. *C. R. Acad. Sci. Paris*, **299**, 575.

RIOU, G., BARROIS, M., DUTRONQUAY, V. & ORTH, G. (1985). Presence of papillomavirus DNA sequences, amplification of c-*myc* and c-Ha-*ras* oncogenes and enhanced expression of c-*myc* in carcinomas of the uterine cervix. In *Papillomavirus: Molecular and Clinical Aspects*, p. 47.

RIOU, G., LE, M.G., LE DOUSSAL, V., BARROIS, M., GEORGE, M. & HAIE, C. (1987). C-*myc* proto-oncogene expression and prognosis in early carcinoma of the uterine cervix. *Lancet*, **i**, 761.

ROTHBERG, P.G., ERISMAN, M.D., DIEHL, R.E., ROVIGATTI, U.G. & ASTRIN, S.M. (1984). Structure and expression of the oncogene c-*myc* in fresh tumor material from patients with hematopoietic malignancies. *Mol. Cel. Biol.*, **4**, 1096.

SCHLUMBERGER, M., CHARBORD, P., FRAGU, P., LUMBROSO, J., PARMENTIER, C. & TUBIANA, M. (1980). Circulating thyroglobulin and thyroid hormones in patients with metastases of differentiated thyroid carcinoma: Relationship to serum thyrotropin levels. *J. Clin. Endocrinol. Metab.*, **51**, 513.

SCHLUMBERGER, M., TUBIANA, M., DE VATHAIRE, F. & 7 others (1986). Long-term results of treatment of 283 patients with lung and bone metastases from differentiated thyroid carcinoma. *J. Clin. Endocrinol. Metab.*, **63**, 960.

TERRIER, PH., DOUC-RASY, S., SCHLUMBERGER, M. & 5 others (1985). Enhanced expression of the c-*myc* oncogene in a case of anaplastic carcinoma of the thyroid gland. In *Thyroid cancer*, Jaffiol, C. & Milhaud, G., (eds) p. 287. Elsevier Science Publishers B.V., Amsterdam.

TRAMONTANO, D., CHIN, W.W., MOSES, A.C. & INGBAR, S.H. (1986). Thyrotropin and dibutyryl cyclic AMP increase levels of c-*myc* and c-*fos* mRNAs in cultured rat thyroid cells. *J. Biol. Chem.*, **261**, 3919.

TUBIANA, M., SCHLUMBERGER, M., ROUGIER, P. & 6 others (1985). Long term results and prognostic factors in patients with differentiated thyroid carcinoma. *Cancer*, **55**, 794.

VERMA, I.M., MITCHELL, R.L., KRUIJER, W. & 4 others (1985). Proto-oncogene *fos*: Induction and regulation during growth and differentiation. *Cancer Cells*, **3**, 275.

YAMASHITA, S., ONG, J., FAGIN, J.A. & MELMED, S. (1986). Expression of the *myc* cellular proto-oncogene in human thyroid tissue. *J. Clin. Endocrinol. Metab.*, **63**, 1170.